# Evaluation of Container Clinics as an Urban Immunization Strategy: Findings from the First Year of Implementation in Ghana, 2017–2018

**DOI:** 10.3390/vaccines11040814

**Published:** 2023-04-07

**Authors:** Anna Shaum, Melissa T. Wardle, Kwame Amponsa-Achiano, Raymond Aborigo, Joseph Opare, Aaron S. Wallace, Delia Bandoh, Pamela Quaye, Fred Osei-Sarpong, Francis Abotsi, George Bonsu, Laura Conklin

**Affiliations:** 1 Center for Global Health, Global Immunization Division, Centers for Disease Control and Prevention, Atlanta, GA 30329, USAccu7@cdc.gov (A.S.W.);; 2 Department of Disease Control and Prevention, Public Health Division, Ghana Health Service, Accra 00233, Ghana; 3 Navrongo Health Research Centre, Health Research Division, Ghana Health Service, Navrongo 03821, Ghana; 4 African Field Epidemiology Network, Kampala 10102, Uganda; 5 Ghana Field Epidemiology and Laboratory Training Programme, School of Public Health, University of Ghana, Accra 00233, Ghana

**Keywords:** vaccines, healthcare access, routine immunization

## Abstract

Background: In 2017, the Expanded Programme on Immunization in Ghana opened two container clinics in Accra, which were cargo containers outfitted to deliver immunizations. At each clinic, we assessed performance and clinic acceptance during the first 12 months of implementation. Methods: We employed a descriptive mixed-method design using monthly administrative immunization data, exit interviews with caregivers of children of <5 years (N = 107), focus group discussions (FGDs) with caregivers (n = 6 FGDs) and nurses (n = 2 FGDs), and in-depth interviews (IDIs) with community leaders (n = 3) and health authorities (n = 3). Results: Monthly administrative data showed that administered vaccine doses increased from 94 during the opening month to 376 in the 12th month across both clinics. Each clinic exceeded its target doses for the 12–23 month population (second dose of measles). Almost all (98%) exit interview participants stated that the clinics made it easier to receive child health services compared to previous health service interactions. The accessibility and acceptability of the container clinics were also supported from health worker and community perspectives. Conclusions: Our initial data support container clinics as an acceptable strategy for delivering immunization services in urban populations, at least in the short term. They can be rapidly deployed and designed to serve working mothers in strategic areas.

## 1. Background

Urbanization is rapidly increasing worldwide; it is estimated that by 2050, 66% of the world’s population will live in an urban area [1]. Much of the increased urbanization is projected to occur on the African continent, where 56% of the population will be urban by 2050 compared to 37% in 2000 [1]. This vast transition to urban living has implications for health systems, and research has indicated significant health and immunization coverage disparities between the poorest urban communities and the wealthiest ones [2,3]. A systematic review of 63 studies conducted in 16 low- and middle-income countries (LMICs) identified that migration status, distance to health facilities, and a lack of parental awareness contribute to the low vaccination status of children living in urban areas [2]. Interventions designed to improve urban immunization coverage in LMICs have primarily focused on community outreach to improve the utilization of services through education, coordinating social mobilization activities, enhancing home visit services, or extending clinic hours [4]. However, urban populations encounter additional barriers to accessing services, highlighting a need for novel strategies to improve access for vulnerable urban communities [2,4].

“Access” can be challenging to define within low-resource contexts in which the overall availability of healthcare is low [5]. Peters et al. (2008) described a framework for accessing healthcare in low-income countries [5] and defined four primary dimensions of access: availability, geographic accessibility, financial accessibility, and acceptability. These dimensions account for the unique experiences in accessing healthcare in LMICs and highlight that the “local adaption and experimentation” of new strategies is critical for improving access. Thus, considering this framework in the initial development and small-scale evaluation of an innovative strategy can improve subsequent implementations and research to enhance access to health services [5].

Immunization services in Ghana are provided at fixed sites (permanent health structures), as well as outreach sites—designated locations where nurses bring vaccine carriers from larger central facilities to deliver immunization services for the day. In comparison to the rural setting, outreach services in many urban settings are frequently not planned or conducted under the assumption that since distance is not as great between fixed sites and households in the urban setting, parents can easily find their way to the existing fixed sites. However, in the urban setting, access to fixed sites can be very challenging due to transport time barriers, transport cost barriers, and busy work schedules. One potential strategy to improve access to immunization in urban settings is the provision of readily accessible, flexible, and convenient, service delivery sites located near urban workplaces or vulnerable urban communities. Container clinics are cargo containers that are converted to clinics and can be used to provide stationary or mobile medical services [6]. These clinics are relatively easy to build, can be easily placed in strategic locations (such as at front of urban markets), and can be adapted to community needs. Container clinics have been utilized to provide infrastructure where health systems are fragile or nonexistent in post-conflict or disaster settings [6,7,8]. However, to our knowledge, none of the published studies have evaluated the feasibility of using container clinics as a strategy to increase the reach of routine immunization services.

In Ghana, as in many LMICs, disparities between urban and rural immunization coverage exist. A study of Ghana’s 2008 and 2014 Demographic Health Surveys (DHS) indicated that children living in urban areas of Ghana were less likely to be fully immunized than children living in rural areas [9]. Furthermore, an analysis of Ghanaian unimmunized children in 2017 found that the urban districts of Kumasi, Accra, and Sekondi-Takoradi had the most unimmunized children [10].

To address the unique urban barriers in accessing immunization services, the Expanded Programme on Immunization (EPI) in Ghana opened two container clinics in the Accra Metropolitan area in September 2017. The clinics were established in nearby locations where nurses previously provided outreach immunization services once per month. They were outfitted to provide daily child health services, including immunizations, home visits, and other forms of preventive and curative care. The container clinics were a small-scale feasibility/demonstration project that fit into a broader initiative known as the Ghana’s Second Year of Life (2YL) project [11]. To inform future scale-up in Ghana and their use in other countries, we evaluated community acceptance and the performance of these container clinics during the first year of implementation.

## 2. Methods

### 2.1. Setting

The two container clinics were established in a large Accra sub-metro area of Ghana, with an overall population of 151,712 (2017 estimate). The sub-metro is divided into five zones that house several health clinics, including a polyclinic, maternity house, and hospital.

**Figure 1:**
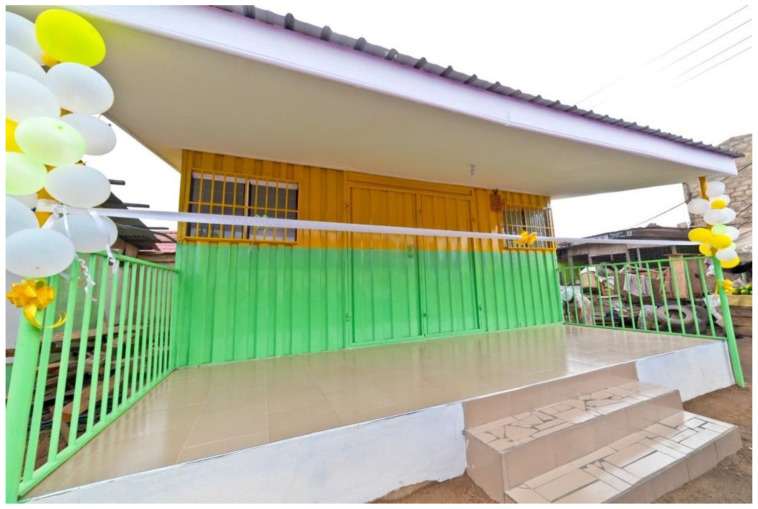
Image of the market container clinic prior to opening in Accra, Ghana, September 2017.

Two of the five zones in the sub-metro were purposively selected container clinic sites. These zones were selected because of expressed community interest, population mobility and vulnerability, limited health infrastructure, and low immunization coverage. For both sites, community advocacy was a key factor in their selection because it is a known indicator of the success of interventions in urban settings [2]. The first container clinic, referred hereafter as the “fishing community clinic,” was placed in a zone with a slum community, directly on the Atlantic coast with a large fishing industry. The second container clinic, referred to hereafter as the “market clinic,” was placed in a zone with a large market in Accra, primarily serving kayayei (informal laborers who carry goods for shoppers at markets) and seasonal migrant workers from the northern part of Ghana. Notably, the selection of this site was also informed by recent research that indicated significant social, cultural, and economic barriers among kayayei women [12,13]. Both clinics were implemented in coordination with the Accra Metropolitan Health Authorities and with the support of community leaders. The clinics were situated on gifted land provided by those same community leaders (see Figure 1 for a picture of the market clinic).

The container clinics were built near the previously designated outreach sites. The two sites planned services for an annual catchment child population of 310 children (0–23 months of age) at the fishing community clinic and 422 children at the market clinic.

### 2.2. Container Clinic Evaluation Design

We used a mixed-method design to evaluate the changes in the immunization services provided at the two locations between September 2017–September 2018. The following information was captured and triangulated to provide initial data on the clinics.

*Infrastructure Assessment*

To describe the evolving infrastructure of the clinics during implementation, we collected data on immunization infrastructure (e.g., cold chain capacity) and the services provided (e.g., vaccines offered) at the sites before or at the time of the clinics’ opening (September 2017), and then six months (March 2018) and 12 months after opening (September 2018).

*Monthly Number of Vaccine Doses*

Every month, we prospectively collected the number of vaccine doses and the type of vaccines administered at each site using the monthly administrative reporting forms (September 2017 to September 2018). We captured the number of vaccine doses administered to children of 0–11 and 12–23 months of age for the pentavalent vaccine (diphtheria-tetanus-pertussis-hepatitis B-Haemophilus influenza type b) (Penta); oral poliovirus vaccine (OPV); measles rubella vaccine (MR), yellow fever vaccine (YF); meningococcal serotype A vaccine (Men A); rotavirus vaccine (Rota); pneumococcal conjugate vaccine (PCV); and inactivated poliovirus vaccine (IPV). Historical administrative records for these sites of when they were outreach posts were largely unavailable, although records from the fishing community site were available for the month prior to opening. Information on target populations for the catchment areas of both clinics were collected from administrative records and the nurses who worked at the outreach vaccination sites. These data were imported, aggregated, and tabulated using Microsoft Excel Office 365 Version 2208.

*Caregiver Exit Interviews*

At 12 months after container clinic implementation (September 2018), we conducted caregiver exit interviews. These were conducted to understand the characteristics of the populations being served and the acceptability of the clinics among the caregivers, and to compare those experiences to the health services they received prior to the clinics’ implementation. We set the convenience sample to a target size of 60 exit interviews per clinic—calculated based on monthly attendance data—or until four weeks of data collection had passed (whichever occurred first). The criteria for participation in the exit interviews included being at least 16 years of age and a caregiver of a child under five. Attending the clinic for immunization was not a criterion for participation.

Information collected from the caregiver exit interviews included demographic variables such as the caregiver’s age (years), child’s age (months), whether or not they were a resident of the community (yes/no), profession (head porter/merchant/hairdresser/seamstress/unemployed/cleaner/other), and educational attainment (none/primary/junior high secondary/senior high secondary/university/unknown). Then, the interviewers proceeded to ask the caregivers about their experiences in accessing services at the container clinics using a structured questionnaire. The questions were aligned with the four dimensions of access including the availability of child health services provided by the container clinics (e.g., the ease of receiving child health care), geographic accessibility (e.g., the convenience of the location, location traveled from, and time spent traveling to the clinic), financial accessibility (e.g., missing work to come to the clinic for child health services), and acceptability (e.g., satisfaction and intention to return). We conducted interviews using tablets with forms programmed in Open Data Kit [14] and uploaded them to SurveyCTO [15].

*Qualitative Focus Group Discussions and In-Depth Interviews*

In September 2018, we also conducted caregiver and nurse focus group discussions (FGDs) and community leader and health authority in-depth interviews (IDIs) at both sites. Caregivers were selected using convenience sampling. The eligibility criteria included living in the catchment area of the container clinic and being a caregiver for a child under five, regardless of if they had attended the clinic. Groups were further stratified based on the caregiver having a child who was 0–23 months old or 24–59 months old. Nurses were eligible if they worked at the clinics before container clinic implementation. Community leaders were invited to participate if they held an authority role in their community and played a key role in setting up the clinic or its operations. Health authorities were selected if they were in a management role or had a unique historical background on the clinic. Guides for FGD and IDIs focused on themes related to access and the group or individual’s perceived change in community access to childhood immunization services.

### 2.3. Statistical and Thematic Analysis

We computed descriptive statistics (counts/percentages, means/standard deviations, or medians/interquartile ranges) for the quantitative variables. The results were examined overall and then stratified by each community area where the container clinics were implemented. We summarized the number of all vaccine doses and the number of MR doses administered each month to monitor changes in the number of vaccine doses administered. For qualitative data, we used deductive codes from the study guides and objectives and added inductive codes as coding progressed. We created themes and sub-themes from the codes generated. We then integrated and organized the data using the conceptual framework described below.

### 2.4. Conceptual Framework

We present our results organized into the dimensions of healthcare access, as described in Peters et al.’s conceptual framework [5]. Recent studies, including those conducted in Ghana and Kenya, have used the framework to understand urban access to care [16,17]. We used the framework’s four dimensions (availability, geographic accessibility, financial accessibility, and acceptability) during the analysis, in addition to the framework’s determinant of ‘individual and household characteristics of users’. Although immunizations are provided free of charge in Ghana, we assessed financial accessibility by examining the direct and indirect costs associated with a clinic visit.

### 2.5. Ethical Considerations

This project was determined to be non-research by the CDC Human Subjects Office, and approval was obtained from the Ghana Health Service’s Ethics Review Committee, as it was evaluated under Ghana’s Second Year of Life project [11]. The purpose of the evaluation was outlined for all participants. Verbal consent was obtained before the data collection, including consent to audio-record the FGDs and IDIs.

## 3. Results

We received monthly administrative immunization data from the clinic sites from September 2017 to September 2018. A total of 107 caregivers participated in the exit interviews across the two sites. We held six caregiver and two nurse FGDs (n= 28 FGD participants) and had IDIs with three community leaders and three health authorities; except for an additional community leader and health authority IDI at the market clinic, an equal number of FGDs and IDIs were held at each site.

### 3.1. Individual and Household Characteristics of Clinic Users

Our findings reflect the different urban populations served at the two container clinics. The exit interview responses revealed that approximately half of the caregivers attending the market clinic lived in the area (n = 25/45; 56%) in contrast to almost all the respondents from the fishing community clinic (n = 61/62; 98%) (Table 1). A higher percentage (13%; n = 8/62) of the caregivers attending the fishing community clinic were unemployed than the market clinic attendees; most women attending the market clinic were traders or head porters (71%; n = 32/45), and none reported unemployment. Additionally, maternal education levels differed at each site; almost half of the market clinic respondents (n = 22/45; 49%) reported receiving either no formal education or only primary school education compared to 20% (n = 13/62) of the fishing community caregivers. Caregivers at the market clinic were of a median age slightly older than that of the caregivers in the fishing community clinic (32 vs. 28 years old). The median age of the children attending was the same across both clinics (12 months).

For the qualitative portion of the evaluation, 34 individuals participated in the interviews and discussions, with 17 participants being from the fishing community, 14 from the market community, and three from the sub-metro health authority.

The average age of caregivers who participated in the FGDs was 29 years for non-attendees and 26 years for attendees. Most participating caregivers were traders (62%) and head porters (19%). The average age of children whose caregivers were in the 0–23-month FGDs was 10.8, while that of children whose caregivers were in the 24- to 59-month group was 33.5 months.

### 3.2. Infrastructure and Availability of Services at Container Clinics

The infrastructure and services provided by both clinics evolved within the first 12 months. Each clinic was developed from an outreach post that provided immunization services once a month by vaccine carriers to an expanded clinic with official EPI reporting tools and EPI refrigerators on-site; full-time staff including 2–6 nurses offered daily routine immunization services. Moreover, at six months, both clinics offered two additional routine vaccines—the rotavirus vaccine (Rota) and pneumococcal conjugate vaccine (PCV)—to their catchment populations, and the newly introduced inactivated polio vaccine (IPV) at 12 months post-implementation (Table 2). This infrastructure improvement was noted during the nurse FGDs:

*…we used to suffer. Before we were doing the outreach without the container, you will be sitting there, sometimes before you even come, the rain has taken all your things, spoilt your registers, dust and all that; so when the container came it was really good.*

—Nurse, the fishing community clinic

*At first, we used to go and carry it (vaccines) from the polyclinic before we come here. And so, by the time the mother will come, the vaccine is not here yet because they come here very early. Now, the moment you come, you just pick your vaccine into your carrier and start working.*

—Nurse, the market clinic

**Table 2:** Summary of services and clinic infrastructure offered by two container clinics pre-implementation, and six months and twelve months post-implementation in the Accra Sub-Metro, 2017–2018.

Services and Infrastructure Offered	Pre-Implementation	6 Months Post-Implementation	12 Months Post-Implementation
Routine immunization services	Outreach	Fixed	Fixed
Frequency of routine immunization sessions	1 session/month	1–2 sessions/week	5 sessions/week
Types of Vaccines	Penta, OPV, MR, YF, Men A	Penta, OPV, MR, YF, Men A, **Rota**, **PCV**	Penta, OPV, MR, YF, Men A, Rota, PCV, **IPV**
Cold chain capacity	Vaccine carrier	Vaccine carrier	Refrigerator Refrigerator tags
Recording and reporting tools	Improvised with notebook Combined with other outreach sites	Improvised with notebook Official immunization recording tools DHIMS	Official immunization recording tools DHIMS
Staff	1–3 staff	2–6 staff	2–6 staff

Bold signifies new vaccines being offered at clinics. Abbreviations: Penta vaccine (diphtheria-tetanus-pertussis-hepatitis B-Haemophilus influenza type b vaccine); OPV (oral polio vaccine); MR (measles rubella vaccine), YF (yellow fever vaccine); Men A (Meningococcal Serotype A vaccine); Rota (rotavirus vaccine); PCV (pneumococcal conjugate vaccine); IPV (inactivated polio vaccine); DHIMS (district health information management system).

**Figure 2:**
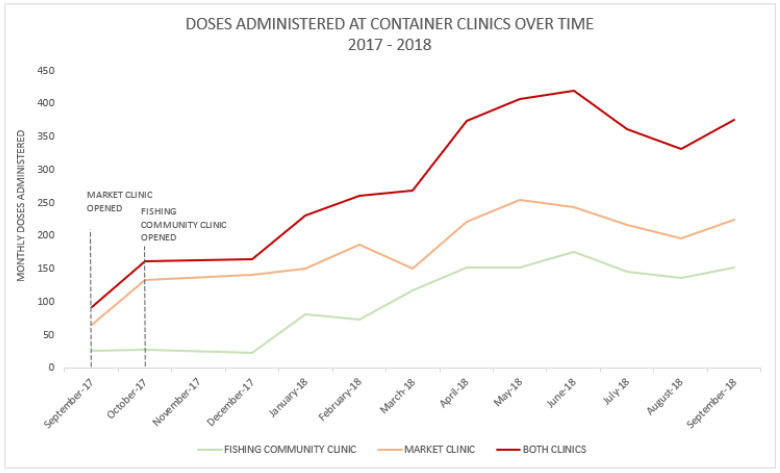
Panel line graph displaying total vaccine doses administered in two container clinics providing immunization services in Accra, Ghana, September 2017–September 2018. Total doses include Penta1-3, PCV1-3, Polio1-3, Rota1-2, MR1, YF, MR2, MenA, and IPV (introduced in June 2018). Abbreviations: “Penta” or Pentavalent vaccine (diphtheria-tetanus-pertussis-hepatitis B-Haemophilus influenza type b vaccine); OPV (oral poliovirus vaccine); MR (measles rubella vaccine), YF (yellow fever vaccine); Men A (Meningococcal Serotype A vaccine); Rota (rotavirus vaccine); PCV (pneumococcal conjugate vaccine); IPV (inactivated poliovirus vaccine).

The increase in the availability of services was matched by an increase in utilization. Over the first year, the number of monthly vaccine doses administered increased consistently. When comparing the first to last month of doses administered, we observed a 442% increase at the market clinic (28 to 152 doses) and a 239% increase at the fishing community clinic (66 to 224 doses). This change included doses of IPV (introduced in June 2018) and the newly offered PCV and Rota (Figure 2).

**Figure 3:**
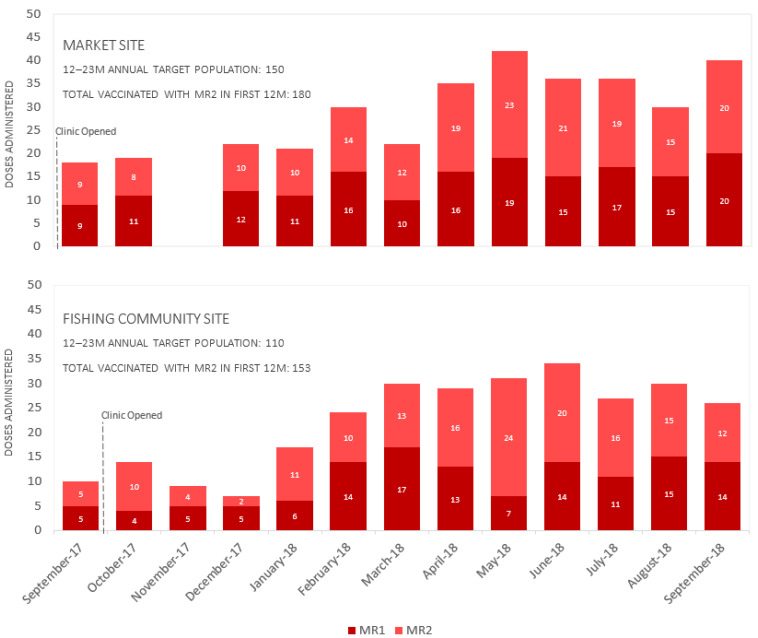
Stacked bar graphs displaying doses administered over time for measles-rubella-1 (MR1) and measles-rubella-2 (MR2) at both container clinic sites from 2017–2018. Abbreviations: MR1 (measles rubella vaccine dose 1); MR2 (measles rubella vaccine dose 2). Note: missing data for the market site in November 2017.

When assessing measles-rubella dose 1 (MR1) and MR2 specifically, both container clinics exceeded their annual target population for MR1 and MR2. For example, at the market clinic, the target population for 12–23 months was 150 children, and 180 doses of MR2 were administered. At the fishing community clinic, the target population for 12–24 months was 110 children, and 153 doses of MR2 were administered (Figure 3). The nurses also described an increase in vaccine uptake among the target population and improved immunization service utilization for traditionally underserved populations:

*It has really increased because we are actually now getting more than what we used to get. Like now, what we get in a week is more than the number we used to get for a month.*

—Nurse, the fishing community clinic

*…let’s take the kayayei, they sleep here unless festive seasons before they go to their places but some people too those who are from northern region, their vaccination is very poor because sometimes a five year old hasn’t taken Penta 3, no vitamin A at 6…when you go for visit you can look out for them and give them the vaccines.*

—Nurse, the market clinic

### 3.3. Geographic and Financial Accessibility of Clinics

On average, it took caregivers (N = 107) five minutes to reach the clinic, with the majority (71%; n = 32/45) in the market community traveling to the clinic from work, and the majority (90%; n = 56/62) in the fishing community coming from home. Almost all (99%; N = 106/107) caregivers said that the container clinic was in a convenient location. The theme of geographic accessibility also emerged from the qualitative data:

*But this container clinic here, excuse me to say, it has helped me a lot, because I have a child that is 3 years, but I still bring him for weighing, now the container services, it is close to our houses.*

—Caregiver, the fishing community

*…I won’t risk this for anything so I will try to always take my child but at first, I always felt lazy to go because I was always here [at the market] so going to the community clinic… but now that this one is closer, why not? I can even send someone to bring the child.*

—Caregiver, the market community

The container clinics appear to improve the financial “accessibility” of receiving an immunization, as almost all caregivers walked to the clinics (97%; N = 104/107) in an average of five minutes (see above). Additionally, among caregivers who did not miss work to attend the container clinics, 59% (n = 23/45) of the caregivers interviewed at the market clinic reported they would have missed work without the container clinic, and 25% (n = 9/62) of the caregivers at the fishing community clinic reported they would have missed work (Table 1). This finding was also seen in the FGDs and IDIs:

*And then the immunization and everything goes on here so it has reduced their transportation in a way so it is a benefit to them, so they don’t complain of not having money to go to the clinic again.*

—Nurse, the market clinic

*…I think that, those that take cars to far places before or walk far distance before going to the clinic has reduced. There is someone who also has to walk before going, she will get tired, container clinic has saved all that hassle.*

—Community leader, the fishing community

### 3.4. Acceptability of Clinics

Approximately 96% (n = 43/45) of caregivers interviewed at the market clinic and 87% (n = 54/62) of caregivers interviewed at the fishing community clinic reported that they planned to return for future services. Additionally, 98% (N = 105/107) of exit interviewees stated that the container clinics made it easier to receive child health services. The top reasons mentioned for why services were easier to receive included: “the clinic is easier to get to than other clinics” (98%), “more suitable hours” (27%), and “immediate attention” (22%). Over three-quarters (86%; N = 92/107) of the caregivers across both sites reported that the container clinic services were either better or equal to those received at other clinics (Table 1).

Despite the high levels of acceptability among the exit interviewees, caregivers reported the need for more services. Among the market clinic caregivers, 24% (n = 14/45) reported that the clinics should provide antenatal care (ANC) services. Of the caregivers attending the fishing community clinic, 23% (n = 14/62) also suggested the addition of ANC services and many recommended that the clinic adds prescription services (40%; n = 25/62). Among the nurses, language barriers were also noted, which may affect the acceptability of services. The desire for expanded services and the challenge of language barriers were corroborated in the qualitative findings:

*So since it is a clinic I think if it is expanded a bit, bring in more workers and increase the facilities so that it won’t be a small place that only dishes out para [cetamol] for a headache then you have to go again to Polyclinic; but it should be a permanent place where if I have stomach pains I can be treated, admitted and if I need infusion I should be given so, if it is expanded a bit it will help us.*

—Caregiver, the market community

*Language barrier because most of them are from these French countries and the north. So they don’t speak English and they don’t understand the Twi too unless their hometown language or sign language and when you do the sign language they understand or sometimes they come along with other people who understand the Twi and their language.*

—Nurse, the market clinic

## 4. Discussion

Our results suggest container clinics are an acceptable strategy to improve access to routine immunization services for the two urban communities in Ghana, and possibly in other similar urban areas. Following Peters et al.’s (2008) conceptual framework [5], we found that the placement of the container clinics within these communities addressed the availability, geographic and financial accessibility, and acceptability of routine immunization services. During the first 12 months of implementation, immunization sessions increased from monthly (when the clinics were only outreach sites) to daily; all recommended vaccines and supporting supplies and tools were stored on-site, and caregivers reported high satisfaction with both clinics. Improvements in geographical and financial accessibility were observed by minimizing commuting distance, time, and the indirect costs of lost wages for caregivers.

Implementing the container clinics required transforming the locations from outreach posts to fixed routine immunization sites. Transforming the sites into functioning clinics was essential because the outreach sites where the container clinics were built would cancel services due to poor weather that impacted vaccination sessions. Lack of resources, supplies, and tools are well-documented barriers to fully vaccinating children and also have implications for building trust with caregivers to return for future vaccination services, as well as the ability of health workers to effectively trace defaulters [17,18,19,20].

The utilization of vaccination services gradually increased as the container clinics became more established over the 12 months. In addition to the three-fold increase in vaccine doses administered during the first 12 months, both clinics exceeded their annual target population for the administration of MR1 and MR2 vaccine doses. Vaccine utilization is driven by a combination of demand and access factors, which this evaluation was not designed to disentangle. However, it is important to note that, in other studies, strategies for increasing demand include improving service frequency, design, and delivery [17,18,19,21]—factors that the container clinics addressed. Thus, we hypothesize that increased community demand for services due to the availability of a geographically proximate, brand new clinic was likely a key factor underlying the observed increase in the utilization of services at these sites.

While most studies have assessed geographical accessibility in the rural poor, recent studies have identified how urban caregivers experience limited access, especially when the distance to the health facility is more than 1–2 km from their home [20,22]. We found that container clinics can provide immunization services to previously hard-to-reach urban populations—nearly every exit interviewee stated that the clinic made receiving care for their child easier compared to accessing previous health service sites. Most caregivers from the exit interviews reported that they walked to the clinics, and data from the caregiver FGDs indicated that the proximity of the clinics was favorable.

Previous studies have found that employed caregivers faced higher opportunity costs when bringing their children to the clinic for vaccinations because of the loss of potential daily earnings [9,20,21,23,24]. In our evaluation, the market community was chosen to provide services to a notably vulnerable group, the kayayei, or head porters. Typically, these head porters are young, rural-urban migrants from the northern part of Ghana [12,25,26] who may forgo accessing health services due to cultural discrimination [26] or because of financial barriers related to lost wages [12]. Our results showed that the majority of the caregivers at the market clinic were indeed head porters or other migrant traders. Interestingly, more market caregivers reported they would have had to miss work if not for the container clinic than mothers in the fishing community did. Thus, placing container clinics near markets may address unique barriers faced by working caregivers, including head porters and migrant traders.

Finally, our results support the growing literature on how the urban poor are not a monolith and require tailored urban immunization strategies to achieve success [2,4,16,21,27]. The fishing community clinic served a stationary population with higher levels of unemployment and education, while the market clinic served a highly mobile population of working mothers with very little education. These differences influenced not only caregiver expectations of the clinic but also the overall acceptability of the clinic; we observed an almost 10% difference in acceptability when caregivers were asked if they planned to return to the container clinic or seek services elsewhere, with more market caregivers indicating they planned to return.

Acknowledging the diversity of urban contexts is important because although timely, the current focus on urban health is in danger of overlooking the nuances and unique challenges faced by the multitude of urban populations in Africa. A recent paper highlighting the diverse urban contexts in Johannesburg also argues that ‘place matters’:

*Whilst the challenge of addressing the health of urban populations within developing countries is acknowledged, the diverse urbanisation experiences of different urban groups remain under-explored…for action to improve the health of poor urban populations to be successful, urban policy makers and programmers need to understand the complexity of the urban context*

[28]

## 5. Limitations

This evaluation had several limitations, including the method of convenience sampling in the exit interviews and FGDs, as well as the small size of the clinics’ catchment populations, which impacted our ability to make inferences about caregivers who were not utilizing the clinics with the exit interview data. To overcome these limitations, we sought to increase the richness of the data by integrating our qualitative and quantitative findings. Additionally, exit interviews are subject to response bias, which can shift responses toward positive feedback. We also relied on administrative records to monitor changes in immunization doses administered over time, which may subject those results to the limitations of written and improvised records in low-resource settings [29]. Finally, while the results provide initial evidence about the success of container clinics in improving urban access to immunization, they are not generalizable to other urban settings in Ghana. We suggest that the lessons learned from this initial implementation inform future scale-up and full impact evaluations of container clinics as an urban immunization strategy.

## 6. Conclusions

Our initial data support that container clinics were an acceptable method for delivering immunization services to urban populations, at least in the short term. Our findings also highlight the importance of community engagement and context-tailored strategies to improve urban access to immunization; container clinics may be a more acceptable strategy when designed to serve working mothers and built-in strategic areas (e.g., urban markets). Further studies to understand the potential role of container clinics as an urban immunization strategy are needed, including studies on their cost-effectiveness, long-term sustainability, impact on immunization coverage, and ability to expand services to meet other community needs.

## Figures and Tables

**Table 1:** Exit interview responses among caregivers of children 0–5 years of age from container clinics in two urban communities on the accessibility and acceptability of services offered 12 months post-implementation, September 2018 (N = 107).

		Total		Market Community		Fishing Community	
		N = 107		n = 45		n = 62	
Exit Interview Question	Response	N	%	n	%	n	%
Do you live in this community?	Yes	86	80.4	25	55.6	61	98.4
	No	21	19.6	20	44.4	1	1.6
What is your profession?	Head porter, trader, merchant	57	53.3	32	71.1	25	40.3
	Hairdresser	9	8.4	2	4.4	7	11.3
	Seamstress	9	8.4	3	6.7	6	9.7
	Unemployed	8	7.5	0	0.0	8	12.9
	Cleaner	5	4.6	1	2.2	4	6.5
	Other *	19	17.8	7	15.6	12	19.3
What is the highest level of education for this child’s mother?	None	22	20.6	16	35.6	6	9.7
	Primary	13	12.1	6	13.3	7	11.3
	Junior High Secondary	51	47.7	18	40.0	33	53.2
	Senior High Secondary	19	17.8	5	11.1	14	22.6
	University	1	0.9	0	0	1	1.6
	Unknown	1	0.9	0	0	1	1.6
Is this location convenient for you?	Yes	106	99.1	44	97.8	62	100
	No	1	0.9	1	2.2	0	0
Did you come here today from home, work, or another location?	Home	69	64.5	13	28.9	56	90.3
	Work	28	26.2	23	51.1	5	8.1
	Home and work are the same	10	9.3	9	20	1	1.6
Did you have to miss work today to come to this container clinic?	Yes	19	17.8	5	11.1	14	22.6
	No	75	70.1	39	86.7	36	58.1
	DK/NA	13	12.1	1	2.2	12	19.3
If no, and this container clinic was not here, would you have had to miss work to get care today? ^	Yes	32	42.7	23	59.0	9	25.0
	No	40	53.3	15	38.5	25	69.4
	DK	3	4.0	1	2.5	2	5.6
Has the new container clinic made it easier to receive medical care for your child/children?	Yes	105	98.1	44	97.8	61	98.4
	No	0	0.0	0	0.0	0	0.0
	DK	2	1.9	1	2.2	1	1.6
If yes, in what ways has it made it easier? ^**	Container clinic is easier for me to get to than other clinics	98	93.3	40	90.9	58	95.1
	The hours are more suitable for my schedule	27	25.7	11	25.0	16	26.2
	Immediate attention	23	21.9	10	22.7	13	21.3
	No charge for service	2	1.9	2	4.5	0	0.0
	Other	5	4.8	1	2.3	4	6.6
How do services you received at clinics before compare to services you receive at the container clinic?	Container clinic is better	40	37.3	18	40.0	22	35.5
	Services are about the same	52	48.6	19	42.2	33	53.2
	Other clinic(s) were better	2	1.9	1	2.2	1	1.6
	Don’t Know	13	12.2	7	15.6	6	9.7
When you or your child need health services in the future, do you plan to return to this container clinic or go to a different clinic?	Return to the container clinic	97	90.7	43	95.6	54	87.1
	Go to a different clinic	6	5.6	1	2.2	5	8.1
	Don’t Know	4	3.7	1	2.2	3	4.8
What recommendations do you have to improve the container clinic? **	No recommendations	41	38.3	28	62.2	13	21.0
	Add prescription services	29	27.1	4	8.9	25	40.3
	Add ANC services	25	23.4	11	24.4	14	22.6
	Expand size of clinic and seating	10	9.3	1	2.2	9	14.5
	Add other services	8	7.5	0	0.0	8	12.9
	Staff a physician/doctor	7	6.5	0	0.0	7	11.3
	Improve advertising	6	5.6	5	11.1	1	1.6

* Other responses included laborer, cook, housewife, artisan, apprentice, sanitation worker, teacher waitress, laundry worker, pedicurist, and binding shop worker. ^ Denominators are determined by responses to a preceding question. ** Responses may exceed sample sizes as participants could provide more than one answer. Abbreviations: Do not know (DK), not applicable (NA).

## Data Availability

The datasets used and/or analyzed during the current project may be available upon reasonable request.
